# Radiation-induced signaling pathways that promote cancer cell survival (Review)

**DOI:** 10.3892/ijo.2014.2614

**Published:** 2014-08-20

**Authors:** ASHLEY L. HEIN, MICHEL M. OUELLETTE, YING YAN

**Affiliations:** Eppley Institute for Research in Cancer and Allied Diseases, University of Nebraska Medical Center, Omaha, NE, USA

**Keywords:** radiation therapy, signaling pathways, cell cycle checkpoint, DNA repair, cell survival

## Abstract

Radiation therapy is a staple cancer treatment approach that has significantly improved local disease control and the overall survival of cancer patients. However, its efficacy is still limited by the development of radiation resistance and the presence of residual disease after therapy that leads to cancer recurrence. Radiation impedes cancer cell growth by inducing cytotoxicity, mainly caused by DNA damage. However, radiation can also simultaneously induce multiple pro-survival signaling pathways, such as those mediated by AKT, ERK and ATM/ATR, which can lead to suppression of apoptosis, induction of cell cycle arrest and/or initiation of DNA repair. These signaling pathways act conjointly to reduce the magnitude of radiation-induced cytotoxicity and promote the development of radioresistance in cancer cells. Thus, targeting these pro-survival pathways has great potential for the radiosensitization of cancer cells. In the present review, we summarize the current literature on how these radiation-activated signaling pathways promote cancer cell survival.

## 1. Introduction

As a staple approach for cancer treatment, radiation therapy plays a critical role in local disease control. When combined with chemotherapy (i.e., chemoradiation), radiation provides additional benefits, which give better disease control and significantly improve cancer patient survival (1–3). However, radioresistance and the presence of residual disease after radiation therapy remain major problems that result in the loss of the therapy effectiveness (4–7). Currently, there is no clinical approach available either for predicting the benefit of radiation therapy for individual cancer patients or for radiosensitization of cancer cells. Thus, an improved understanding of the mechanisms that promote cancer cell survival after radiation could allow pharmacological strategies to be developed to improve the efficacy of radiation therapy.

Radiation exposure induces numerous cellular signaling pathways, which can lead to cellular responses including apoptosis, cellular senescence and cell cycle checkpoint activation/DNA repair (8). Among the radiation-induced pro-survival signaling pathways, some are involved in inducing cell cycle arrest and promoting DNA repair, while others are engaged in suppressing apoptosis induction (9,10). These pathways act synergistically to protect cancer cells from the cytotoxic effects of radiation, ultimately leading to the development of radioresistance. This review summarizes the signaling pathways that positively contribute to cancer cell survival in response to ionizing radiation.

## 2. HER (also called ERBB or EGFR) signaling

**Figure 1:**
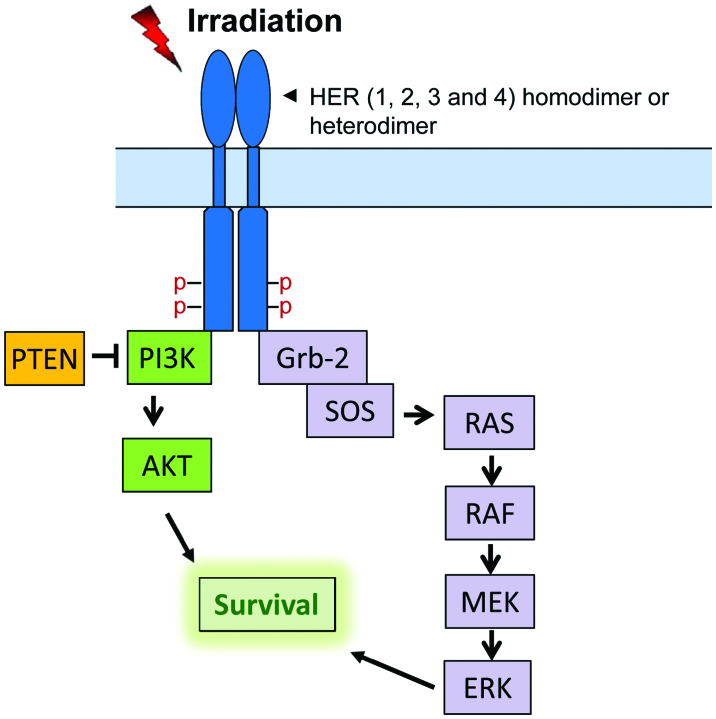
Radiation induces activation of HER receptors, which, in turn, leads to the activation of PI3K/AKT and RAS/RAF/MEK/ERK signaling pathways that promote cell survival.

The HER family of receptor tyrosine kinases (RTKs) consists of HER1, HER2, HER3 and HER4, which localize on the cell membrane (11). HER RTKs share a similar protein structure that contains an extracellular region (ligand binding and dimerization domains), a transmembrane region and an intracellular region (protein tyrosine kinase domain and phosphorylation regulatory tail) (12). Among HER receptors, HER2 has no known ligand and HER3 possesses very low kinase activity (12). Binding of ligands to the ligand binding domain of HER1, HER3 and HER4 results in homo- or hetero-dimerization of the receptors followed by trans-phosphorylation of several tyrosines in the c-terminal regulatory tail of the receptor (12). The phosphorylated tyrosines form docking sites for downstream adaptors and signal transducers, activating downstream signaling pathways including PI3K/AKT, RAS/RAF/MEK/ERK, phospholipase C-γ/protein kinase C and JAK/STAT pathways (13,14). Among those pathways, PI3K/AKT and RAS/RAF/MEK/ERK cascades have been shown to play important roles in cell survival after radiation (Fig. 1) (15).

An increase in HER1 phosphorylation, indicative of HER activation, following ionizing radiation has been reported previously (16–18). Our most recent study in human breast cancer cells demonstrates that ionizing radiation results in an increase in phosphorylation of not only HER1, but also HER2, HER3 and HER4 (19). Although the mechanisms responsible for this phosphorylation of HER receptors has not yet been determined, previous studies have shown that receptor protein tyrosine phosphatases (PTPs), which suppress HER RTK phosphorylation, can effectively be inhibited by reactive oxygen/nitrogen species (ROS/RNS) through oxidation (20). Previous studies have also demonstrated that radiation induces ROS/RNS production via a mitochondria-dependent mechanism (21). Thus, the ROS/RNS production in response to radiation could lead to the inhibition of PTPs, resulting in the activation of HER RTKs. Future studies will be needed to examine this possibility for the activation of HER RTKs following radiation.

Inhibition of HER RTKs has been shown to increase the radiosensitivity of cancer cells. Inhibition of HER RTKs by HER pan-inhibitor CI-1033 notably enhances the radiosensitivity of human colon carcinoma cells both *in vitro* and *in vivo* (22), while HER1 inhibition by gefitinib and HER2 inhibition by herceptin, respectively, radiosensitizes EGFR amplified glioma cells and breast cancer cells (23,24). Generally, the pro-survival function of HER receptors involves at least two possible mechanisms. The first mechanism is based on the capability of HER receptors to activate AKT and ERK1/2 signaling, which play critical roles in suppressing apoptosis (15). Another possible mechanism for the pro-survival function of HER receptors is through their regulation of the cell cycle checkpoint response and DNA repair. In our recent study, we found that HER2 activation following radiation is necessary for the activation of the G2/M cell cycle checkpoint response (19). In addition, HER1 has been reported to promote the activation of DNA-dependent protein kinase (DNA-PK), which plays an essential role in the NHEJ-mediated repair of DNA double-strand breaks (DSBs) (25,26).

## 3. Extracellular signal-regulated kinase (ERK1/2) pathway

In a wide variety of cell types, ionizing radiation induces rapid activation of MAPK family members, including ERK1/2, JNK and p38 (27,28). Among those, radiation-induced ERK1/2 signaling activation has been shown to play an important role in promoting cell survival in response to radiation (29–31).

Following radiation, ERK1/2 is activated through dual tyrosine and threonine phosphorylation by MEK1/2 and the activation, in turn, leads to the phosphorylation/activation of over 160 substrates (32). Some of these substrates are transcription factors that regulate the expression of genes encoding for anti-apoptotic proteins (27,32). The best characterized antiapoptotic transcription factors targeted by ERK1/2 signaling are the cyclic AMP-responsive element binding protein (CREB) and CAAT/enhancer binding protein β (C/EBP-β). In response to radiation, ERK1/2 phosphorylates/activates p90^rsk^ kinase, which in turn activates CREB and C/EBP-β, thereby inducing the expression of a number of anti-apoptotic proteins including Bcl-xL, Mcl-1 and c-FLIPs (33–35). In addition, ERK1/2 can directly phosphorylate and inhibit a number of pro-apoptotic proteins, including Bad, Bim and caspase 9 (36–39). Thus, by increasing the expression/activity of anti-apoptotic proteins and inhibiting the activity of pro-apoptotic proteins, the net effect of the radiation-induced ERK1/2 signaling activation is the suppression of apoptosis in irradiated cells.

Studies from our group and others have demonstrated that ERK1/2 signaling activation after radiation is essential for activation of the G2/M cell cycle checkpoint in response to radiation (29,31,40–42). Radiation-induced ERK1/2 signaling is required for the activation of key regulators of the G2 checkpoint, most notably ATR and BRCA1 (31,42). ERK1/2 signaling also plays an important role in promoting DNA repair. Radiation-induced ERK1/2 signaling has been associated with the transcriptional upregulation of genes involved in DNA repair, such as *ERCC1, XRCC1* and *XPC* (43,44). Furthermore, ERK1/2 signaling can activate DNA-PK, which plays a critical role in NHEJ-mediated DSB repair, and PARP-1, which recognizes single-stranded DNA breaks (SSBs) on the damaged DNA (44–47). Also, ERK1/2 signaling functions as a positive regulator of ataxia telangiectasia mutated (ATM)-dependent homologous recombination (HR) DSB repair (48). Thus, by promoting G2/M cell cycle checkpoint activation and increasing DNA repair, ERK1/2 signaling positively regulates cancer cell survival following radiation. Consistent with these observations, an increasing number of studies demonstrate that constitutive activation of Ras results in an increase in the radioresistance of cancer cells, whereas inhibition of MEK or ERK leads to the radiosensitization of cancer cells (29,40,41,49).

While the exact mechanisms responsible for the activation of ERK1/2 signaling by radiation has not yet been clearly elucidated, several signaling mechanisms have been proposed to be involved in this activation. As demonstrated by us and others, the rapid activation of HER family receptors following ionizing radiation contributes to ERK1/2 signaling activation in cancer cells of the breast and lung (17). Furthermore, this role of HER receptors involves Ras GTPase. An activation of Ras in response to HER receptor activation (mainly HER1 and HER2) has been demonstrated and ectopic expression of Ras-N17 dominant negative mutant abolishes the ERK1/2 activation by radiation (50,51). Via recruitment of Grb-2 to the activated HER receptors, Grb-2 becomes activated and forms a complex with SOS protein, which triggers the activation of Ras/Raf/MEK/ERK signaling (Fig. 1) (50,51). Furthermore, the activated Ras can induce HER1-ligand production, which, through an autocrine feedback loop, further activates HER1 and then Ras/Raf/MEK/ERK signaling (52,53). Another mechanism implicated in radiation-induced ERK1/2 activation involves the tumor suppressor BRCA1. Studies from our laboratory show that decreasing BRCA1 expression in breast cancer cells using shRNA markedly diminishes the activation of ERK1/2 signaling after radiation (42). Conversely, inhibition of ERK1/2 signaling using pharmacological inhibitors or siRNA also results in the destabilization of BRCA1 protein in irradiated breast cancer cells (42). These results suggest a positive feedback loop involving ERK1/2 and BRCA1 in response to ionizing radiation. Lastly, the DNA damage sensor ATM has also been implicated in radiation-induced ERK1/2 activation (48). ERK1/2 activation following radiation has been shown to require ATM, as ATM inhibition partially blocks the radiation-induced ERK1/2 activation (48). Conversely, inhibition of ERK1/2 signaling can also attenuate radiation-induced ATM phosphorylation, as well as the recruitment of ATM to DNA damage foci (48). These studies suggest another positive feedback loop in the radiation response, this time involving ATM and ERK1/2. Collectively, these studies indicate that the activation of ERK1/2 signaling in response to radiation is regulated by multiple inter-regulated signaling pathways.

## 4. AKT signaling pathway

**Figure 2:**
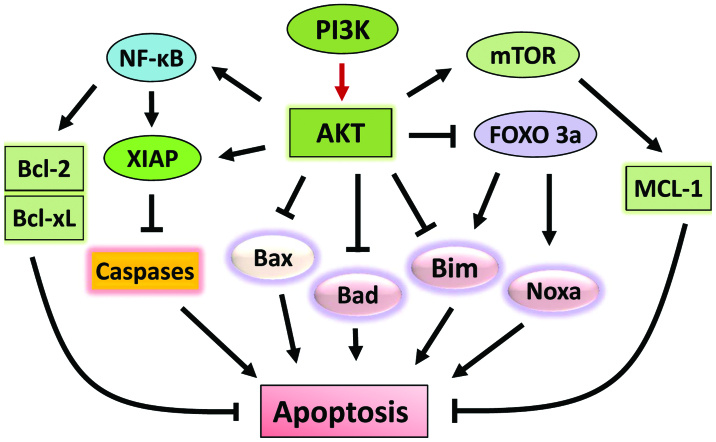
PI3K/AKT mediated signaling promotes cell survival. i) Activation of PI3K by radiation leads to the phosphorylation/activation of AKT; ii) AKT phosphorylates and inhibits pro-apoptotic proteins Bad, Bax, Bim and Noxa; iii) AKT phosphorylates and activates pro-survival transcription factor NF-κB, leading to the upregulation of pro-survival genes *BCL-2* and *BCL-XL*; iv) AKT phosphorylates pro-survival protein XIAP, which binds and inhibits caspase 3/7/9, which are required for apoptosis induction; v) AKT phosphorylates/activates mTOR kinase, which phosphorylates/activates antiapoptotic protein Mcl-1; vi) FOXO3a upregulates the gene expression of pro-apoptotic proteins Bim and Noxa. Phosphorylation of FOXO3a by AKT results in inhibition and nuclei exclusion of the protein.

The AKT signaling pathway plays a vital role in cell survival. Aberrant activation of this signaling cascade has been detected in various types of malignancies and is associated with tumorigenesis (54). AKT functions as a pro-survival factor by inhibiting apoptotic signal cascades and activating pro-survival signaling pathways (Fig. 2). Upon activation, AKT phosphorylates and inhibits a number of pro-apoptotic Bcl-2 family members, including Bad, Bax and Bim (55–57). Furthermore, through direct inhibition and exclusion of proapoptotic transcription factor FOXO3a (Forkhead box O3), AKT also suppresses the expressions of the pro-apoptotic factors Bim and Noxa (58–61).

AKT also positively regulates anti-apoptotic pathways (Fig. 2). AKT induces activation of NF-κB transcription factor, which promotes the transcription of a wide range of anti-apoptotic genes, in particular *BCL-2* and *BCL-XL* (62). Furthermore, AKT phosphorylates/activates pro-survival protein XIAP (X-linked inhibitor of apoptosis protein), resulting in an increase of binding of XIAP to caspases 3, 7 and 9 and inhibition of these caspases, the activities of which are essential for apoptosis induction (63). Another key pro-survival pathway targeted by AKT is the mTOR signaling pathway. AKT phosphorylates and activates mTOR kinase, leading to the phosphorylation/activation of anti-apoptotic protein Mcl-1 (64,65). Furthermore, AKT negatively regulates hypoxia-induced apoptosis. Following radiation therapy, hypoxia is often induced in tissues by radiation and can lead to apoptosis in the injured tissue (66,67). The hypoxia-induced apoptosis requires glycogen synthase kinase (GSK) to activate the mitochondria-dependent death signaling pathway (67,68). However, AKT activation following radiation can inhibits the activity of GSK through phosphorylation, resulting in an activation of glycolysis and glucose transport that suppresses apoptosis induction (69). Lastly, AKT is directly involved in the activation of the catalytic subunit of DNA-PK after radiation, promoting NHEJ-mediated DSB repair that increases cell survival (70). These studies establish a pro-survival role for AKT mediated signaling pathways in the response of cancer cells to radiation.

Activation of the PI3K/AKT signaling pathway in response to ionizing radiation has been widely observed (15). A likely mechanism for this activation involves HER RTKs. Upon activation of HER RTKs, the phosphorylated tyrosines in the carboxyl-terminal regulatory tail of HER3 can form six docking sites for recruitment of the p85 adaptor subunit of phosphatidylinositol 3-kinase (PI3K) (71). Subsequently, the p110 catalytic subunit of PI3K phosphorylates phosphatidylinositol-4,5-biphosphate (PIP2) to generate phosphatidylinositol (3,4,5)-triphosphate (PIP3), which then leads to the membrane recruitment and activation of proteins that contain a phospholipid-binding (PH) domain, such as phosphoinositide-dependent kinase (PDK) 1 (72). The activated PDK1 phosphorylates AKT-Thr308 and leads to the initial AKT activation (72). The full-activation of AKT requires further phosphorylation of its Ser473 by PDK2 (72). Furthermore, mutant K-Ras also positively contributes to the activation of PI3K-AKT signaling in response to radiation, which is through its activation of autocrine production of EGFR ligands (73,74).

The pro-survival function of PI3K/AKT signaling is expected to positively contribute to the radioresistance of tumor cells. Indeed, an increasing number of studies indicate that inhibition of PI3K/AKT signaling by either pharmacological inhibitors or genetic approaches leads to an enhancement of radiosensitivity of cancer cells both *in vitro* and *in vivo* (75–77). Furthermore, the increase in radiosensitivity by PI3K/AKT inhibition involves both the diminution of DNA repair and an enhancement of apoptosis induction (70,75,76,78,79). On the other hand, in some cell-based models, inhibition of PI3K/AKT has been shown to have little effect on radiosensitivity (29,80–83), suggesting an involvement of PI3K/AKT-independent mechanisms in the regulation of radiosensitivity.

## 5. Cell cycle checkpoint signaling

**Figure 3:**
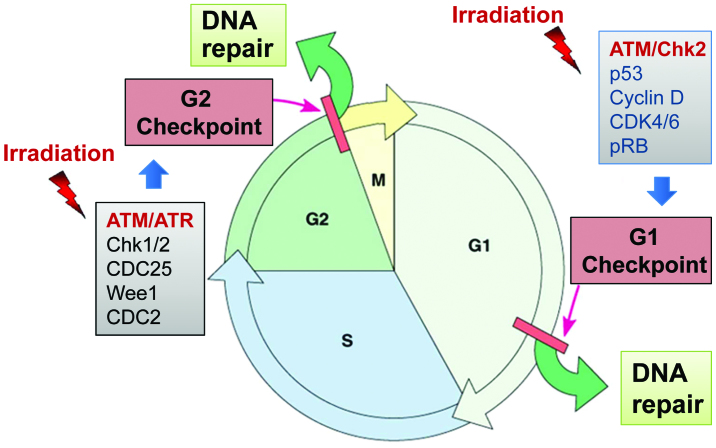
Irradiation induces G1 and G2 cell cycle checkpoint activation and DNA repair. Most cancer cells are defective in G1 checkpoint, commonly due to the mutations/alterations of the key regulators of the G1 checkpoint (in blue), but contain a functional G2 checkpoint.

In response to DNA damage, cell cycle checkpoints often become activated to block cell cycle progression, allowing time for cells to repair the damage (84). Depending on the phase of the cell cycle at which the damage is sensed, the cells can be blocked at the G1/S or G2/M border of the cell cycle (Fig. 3) (84). If the damage is irreversible or the cell cycle checkpoint is dysfunctional, apoptosis may be triggered to eliminate the injured cells (84). Thus, properly functioning cell cycle checkpoints promote cell survival by counteracting the cytotoxicity of DNA damage.

The G1/S transition is controlled by the activity of Cdk4/6 kinases coupled with Cyclin D, the activities of which are predominantly regulated by the p53/p21 pathway (80). The G2/M border is tightly controlled by the Cdc2/Cyclin B complex, whose activity is required for the G2/M transition of the cell cycle (85). The G1 checkpoint is defective in most cancer cells, commonly due to mutations/alterations of key regulators of the G1 checkpoint (p53, Cyclin D, etc.) (80), whereas activation of the G2 checkpoint is rarely impaired in cancer cells, as this checkpoint operates primarily via a p53-independent mechanism (Fig. 3) (86). In fact, in cancer cells lacking a functional G1 checkpoint, abrogation of the G2 checkpoint often sensitizes the cells to radiation (87).

Previous studies identified Cdc2-Y15 as a vital site involved in G2 checkpoint control in response to radiation (88). Cdc2-Y15 is phosphorylated in response to radiation exposure and this phosphorylation is maintained during radiation-induced G2/M arrest (88–90). Cdc2-Y15 is phosphorylated by the Wee1 and Myt1 kinases (91,92) and dephosphorylated by the Cdc25 dual-specificity phosphatases (93).

ATM- and ATR-mediated signaling pathways play essential roles in the radiation-induced cell cycle checkpoint responses (84). In response to radiation-induced DNA-damage, ATM and ATR kinases are rapidly activated, which, in turn, activate their respective downstream targets, including p53 as well as the Chk1 and Chk2 kinases (Fig. 3) (84). Activation of Chk1 and Chk2 results in phosphorylation of Cdc25, leading to the subcellular sequestration, degradation and/or inhibition of the Cdc25 that normally activates Cdc2/Cyclin B at the G2/M boundary (94). Chk2 can also phosphorylate p53-Ser20 to induce stabilization of the p53 protein following radiation (84). Activation of p53 by ATM, ATR and Chk2 kinases leads to the induction of p21 protein, which can directly inhibit the activity of the Cdc2/Cyclin B complex (84).

In summary, radiation-induced cell cycle checkpoint signaling pathways promote cell cycle arrest, which, in turn, contributes positively to cell survival in response to radiation.

## 6. DNA repair pathways

The cytotoxicity caused by ionizing radiation is mainly the result of DNA damage. Radiation induces several forms of DNA damage, which include SSBs, DSBs, sugar and base modifications and DNA-protein crosslinks (95,96). Among these, DSBs are not only a dominant form of damage caused by ionizing radiation (97,98), but also is the most deadly form of DNA damage, as unrepaired DSBs can lead to lethality of cells (97,99).

In response to ionizing radiation, the activation of the phosphoinositide 3-kinase-related kinases (PIKKs), including ATM, ATR and DNA-PK, transduces and amplifies the DNA-damage signal, triggering the assembly of DNA repair apparatuses at the damaged sites and initiating DNA repair (10). A DSB is repaired by one of two competing mechanisms: non-homologous end joining repair (NHEJ) and homologous recombination (HR) (10), with both mechanisms regulated by PIKKs. With no sequence homology required, NHEJ rejoins the free ends in a process that commonly produces errors at the point of junction (100). Each of the two ends is recognized by the Ku70/Ku80 heterodimer, which then recruits DNA-PK (100). Once formed, these complexes bring the ends together for further processing and ligation by DNA ligase IV (100). In contrast to NHEJ, HR repairs DSBs accurately and with very high fidelity (100). This process operates during the S and G2 phases and repairs DSBs taking advantage of sequence information present in the intact sister chromatid (100). Radiation also creates SSBs, mainly through base oxidation driven by ROS/RNS (98). The repair of this type of damage uses base excision repair, which removes the damaged base using a DNA glycosylase and AP endonuclease and then fills up the nick through the actions of DNA polymerases and DNA ligase (101). Consequently, successful DNA repairs promote cell survival in response to radiation, whereas a failure to repair the DNA damage enhances the cytotoxic effect of radiation, leading to lethality of the cells.

## 7. Conclusion

**Figure 4:**
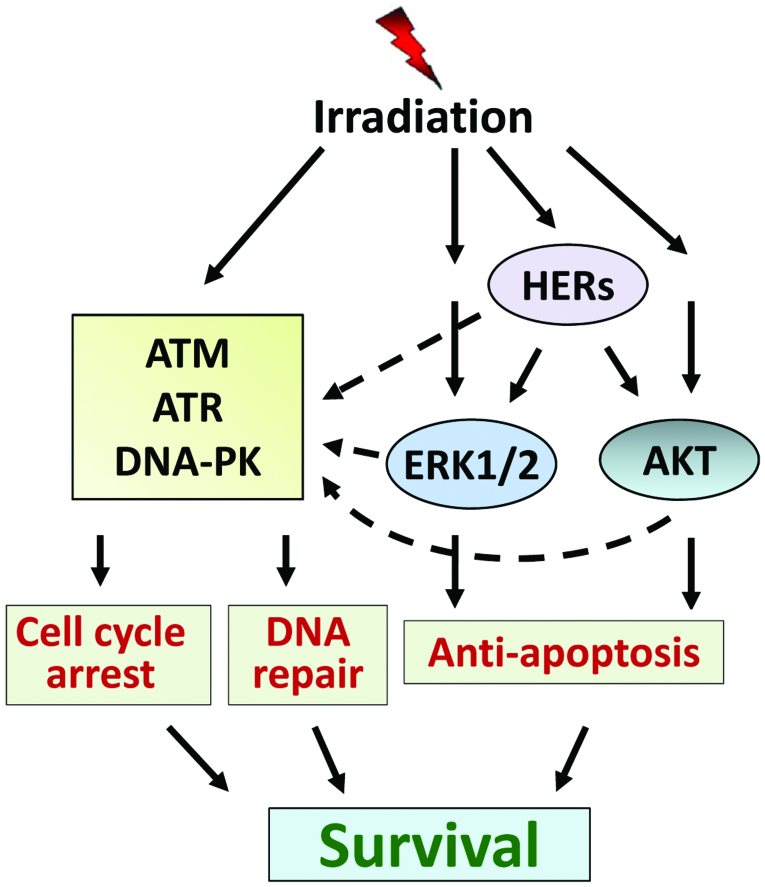
Overview of radiation-induced signaling pathways that promote cell survival. Activation of ATM, ATR and DNA-PK signaling by radiation leads to cell cycle arrest and DNA repair. Activation of HER, ERK1/2 and AKT signaling pathways by radiation suppresses apoptosis induction. HER, ERK1/2 and AKT signaling activation following radiation positively regulate cell cycle checkpoint response and DNA repair.

As a standard of care, radiation therapy plays an important role in cancer therapy. However, radiation resistance remains a major obstacle that limits the efficacy of radiation therapy for cancer treatment. In order to improve the efficacy of radiation therapy, it is necessary that we fully understand the signaling network that drives cancer cells to overcome radiation-induced cytotoxicity, leading to survival. As discussed above, the lethal cytotoxicity caused by ionizing radiation is mainly the result of DSBs. However, radiation also simultaneously induces multiple signaling pathways that can protect cells from the cytotoxic effect of radiation. Among these, signalings mediated by HER receptors, ERK1/2 and AKT prevent the irradiated cells from undergoing apoptosis induction, while signalings mediated by ATM, ATR and DNA-PK drive cells into cycle arrest and initiate DNA repair. In addition, HER ERK1/2 and AKT signaling also positively regulate the cell cycle checkpoint response and DNA repair machinery. Consequently, these signaling pathways act conjointly to rescue the cells from radiation-induced injury and promote survival (Fig. 4). To overcome radiation therapy resistance, future research should focus on the development of pharmacological approaches to block the activation of these pro-survival signaling pathways in irradiated cells.
